# Excessive Accumulation of Intracellular Ca^2+^ After Acute Exercise Potentiated Impairment of T-cell Function

**DOI:** 10.3389/fphys.2021.728625

**Published:** 2021-11-26

**Authors:** Renyi Liu, Karsten Krüger, Christian Pilat, Wei Fan, Yu Xiao, Michael Seimetz, Robert Ringseis, Eveline Baumgart-Vogt, Klaus Eder, Norbert Weissmann, Frank Christoph Mooren

**Affiliations:** ^1^Department of Physical Education, China University of Geosciences (Wuhan), Wuhan, China; ^2^Department of Exercise Physiology and Sports Therapy, Institute of Sports Science, Justus-Liebig-University Giessen, Giessen, Germany; ^3^Institute for Anatomy and Cell Biology II, Justus-Liebig-University Giessen, Giessen, Germany; ^4^Excellence Cluster Cardio-Pulmonary Institute (CPI), Universities of Giessen and Marburg Lung Center (UGMLC), Member of the German Lung Center (DZL), Justus-Liebig-University Giessen, Giessen, Germany; ^5^Institute of Animal Nutrition and Nutrition Physiology, Justus-Liebig-University Giessen, Giessen, Germany; ^6^Department of Rehabilitation Sciences, Faculty of Health, University of Witten/Herdecke, Witten, Germany

**Keywords:** acute exercise, Fura-2(AM), intracellular Ca^2+^, calcium homeostasis, cell proliferation, calcium channels

## Abstract

Ca^2+^ is an important intracellular second messenger known to regulate several cellular functions. This research aimed to investigate the mechanisms of exercise-induced immunosuppression by measuring intracellular calcium levels, Ca^2+^-regulating gene expression, and agonist-evoked proliferation of murine splenic T lymphocytes. Mice were randomly assigned to the control, sedentary group (C), and three experimental groups, which performed a single bout of intensive and exhaustive treadmill exercise. Murine splenic lymphocytes were separated by density-gradient centrifugation immediately (E0), 3h (E3), and 24h after exercise (E24). Fura-2/AM was used to monitor cytoplasmic free Ca^2+^ concentration in living cells. The combined method of carboxyfluorescein diacetate succinimidyl ester (CFSE) labeling and flow cytometry was used for the detection of T cell proliferation. The transcriptional level of Ca^2+^-regulating genes was quantified by using qPCR. Both basal intracellular Ca^2+^ levels and agonist (ConA, OKT3, or thapsigargin)-induced Ca^2+^ transients were significantly elevated at E3 group (*p*<0.05 vs. control). However, mitogen-induced cell proliferation was significantly decreased at E3 group (*p*<0.05 vs. control). In parallel, the transcriptional level of plasma membrane Ca^2+^-ATPases (PMCA), sarco/endoplasmic reticulum Ca^2+^-ATPases (SERCA), TRPC1, and P2X7 was significantly downregulated, and the transcriptional level of IP_3_R2 and RyR2 was significantly upregulated in E3 (*p*<0.01 vs. control). In summary, this study demonstrated that acute exercise affected intracellular calcium homeostasis, most likely by enhancing transmembrane Ca^2+^ influx into cells and by reducing expression of Ca^2+^-ATPases such as PMCA and SERCA. However, altered Ca^2+^ signals were not transduced into an enhanced T cell proliferation suggesting other pathways to be responsible for the transient exercise-associated immunosuppression.

## Introduction

Exercise can enhance marked transient physiological changes and has a profound influence on the human immune function. There are a number of studies showing that the effects of acute exhaustive exercise seem to be detrimental, while regular, moderate-intensity physical activity can improve immune defense functions. There is evidence that the transient “open-window” of immunosuppression might exist in the recovery from strenuous exercise as indicated by an impaired immunity around 3–72h after exercise when athletes seem to be more susceptible to infections. However, this consensus meaning has been disagreed recently due to novel findings demonstrating that exercise stimulates T cell redistribution within organs and tissues, enhances mobilization of hematopoietic stem cells as a result of apoptotic T cells, and reverses T cell immunosenescence (Simpson et al., 2020). Therefore, the effect of a single bout of intensive exercise on immune function remains a controversial topic, and the investigation of underlying molecular mechanisms seems to be a helpful approach to enhance our current understanding.

Ca^2+^ is a key second messenger in the network of cellular signal transduction, which participates in many physiological and pathological processes. The stress of exercise may influence the intracellular Ca^2+^ dynamics of lymphocytes. By using Fura-2 AM dual-wavelength detection technology, our previous work reported that long-term physical training had a significant effect on intracellular calcium signal transduction of lymphocytes in mice (Liu et al., 2017). Since alterations of calcium metabolism have been shown to be involved in some abnormal immune responses, we hypothesized that the mechanisms of acute exercise-induced immunosuppression involve abnormalities in intracellular calcium handling. In order to test this hypothesis, this research examined the effects of acute exercise on intracellular free calcium ion concentrations under basal and agonist-stimulated conditions, the expression of Ca^2+^-handling factors, and changes of T cell function.

## Materials and Methods

### Animals

In this study, we used a total of 60, 12-week-old male CD1 Swiss mice, weighing (28.0±3.2g), which were fed in the animal facilities at the Department of Sports Medicine of University of Münster and Justus-Liebig University (Germany), and kept under controlled conditions of temperature and humidity. This specie and model were chosen for being a homogeneous line of mice. Mice were housed collectively (4–6 per cage) and fed tap standard rodent diet and water at will, with a standard 12h day-night cycle. All procedures were performed following the approval of the local Animal Care and Use Committee. We exert our effort to minimize animal pain and discomfort, and this experiment was performed in accordance with the ARRIVE guidelines.

### Treadmill Incremental Test and Exercise Protocol

Animals were adapted to exercise for 1week before the exercise test, aiming to minimize the stress induced by the equipment. Each animal underwent an incremental exercise test to exhaustion to measure individual maximal oxygen uptake (VO_2_max) and the fastest speed as our group previously described (Kruger et al., 2009). After a 5-min warm-up at 0.20m/s, the running speed was elevated by 0.05m/s every 3min until exhaustion. The animals were randomly divided into in four groups, five in each group. The animals in the control group had exposure to the noise of treadmill and allowed to freely run on a treadmill without effort, while the animals of the exercise groups were submitted a single bout of exercise of 80% VO_2_max workload until exhaustion, which were anesthetized and killed immediately (E0), 3h (E3), and 24h (E24) after exercise for spleen removal.

### Preparation of Lymphocytes From Murine Spleens

After the animals were anaesthetized and killed by cervical dislocation, their spleens were separated under aseptic conditions and placed on a 100-μm-pore size nylon mesh (BD Falcon™ Cell Strainer, BD Biosciences, Heidelberg, Germany). And the mesh was put into a culture dish, which PBS was poured into. The spleen was gently squeezed with a 2-ml syringe plunger to generate single cell suspensions. Biocoll separating solution (Biochrom, Berlin, Germany) was used, and lymphocytes are stratified after density gradient centrifugation as our group described before (Liu et al., 2017). The white band of lymphocytes was removed after centrifugation and washed two times by centrifugation in Hanks’ Balance Salt Solution (HBSS) containing 5% heat-inactivated fetal calf serum (FCS; Gibco, Darmstadt, Germany). The cell suspension in RPMI1640 containing 10% FCS was prepared to be measured. Cell viability (98%) was quantified by the Trypan blue exclusion assay, whereas purity (95%) was verified by a flow cytometry (EPICS XL Beckman Coulter, Fullerton, CA, United States) in the forward/sideward scatter mode. Moreover, the percentage of T cells in the remaining mixed lymphocyte population was determined to be about 85% by labeling with anti-CD3 antibody as described recently (Krüger et al., 2008). The number of cells was then counted by using a semiautomated hematology analyzer (F-820, Sysmex, Norderstedt, Germany).

### Determination of Cytosolic-Free Calcium

Intracellular Ca^2+^ level was assessed by the fluorescence intensity ratio of the calcium probe Fura-2 AM (Molecular Probes Inc., Eugene, OR, United States) as described our previous study (Liu et al., 2017). Briefly, the cells were loaded with 5μl/ml Fura-2 AM stock (1mM in DMSO) in 0.8mM Ca^2+^-containing solution (140mM NaCl, 3mM KCl, 0.4mM Na_2_HPO_4_, 10mM HEPES, 5mM glucose, and 1mM MgCl_2_, and 0.8mM CaCl_2_ with pH 7.4). The fluorescence intensities were measured in the dual wavelength ratio mode in a spectrofluorometer (Deltascan; PTI, Canada) at 340 and 380nm (excitation filters) and 510nm (emission filter). According to the equation of Grynkiewicz et al. (1985), [Ca^2+^]i was calculated as follows: [Ca^2+^]i=(*R*-*R*min)/(*R*max-*R*)×Kd×*F*, with a Kd of Fura-2 for calcium of 220nM, and where *R* is the ratio of fluorescence of the sample at 340 and 380nm. *R*max and *R*min are the ratios for Fura-2 at these wavelengths in the presence of saturating Ca^2+^ (after application of 10mM digitonin) and during Ca^2+^-free conditions (after addition of EGTA, 10mM final concentration), respectively, and *F* is the ratio of fluorescence intensity at 380nm during Ca^2+^-free conditions to the fluorescence intensity at 380nm during Ca^2+^-saturating conditions. To remove the free fura-2AM, cells loaded with the dye were washed two times with PBS solution. The maximum peak and plateau level of the agonist-induced [Ca^2+^]_i_ transient was quantified after the addition of mitogen, concanavalin A (Con A; Sigma Aldrich, St. Louis, United States), the anti-CD3 antibodies (OKT3; Janssen-Cilag, Neuss, Germany), or thapsigargin (TG; Sigma Aldrich, St. Louis, United States). To estimate intracellular Ca^2+^ release and extracellular Ca^2+^ influx, experiments were performed either in absence or in presence of 0.8mmol/l Ca^2+^ in the measurement medium. For experiments performed in the absence of extracellular calcium, CaCl_2_ was replaced by 1mM EGTA in the buffer. The addition of Ca^2+^ allowed measuring transmembrane Ca^2+^ influx.

### Mn^2+^-Quenching Experiments

Bivalent cation, Mn^2+^ and Ca^2+^, can share common entry channels in cell membrane (Sage et al., 1989), and the former can bind to intracellular Fura-2 (Fura-2-acetoxymethyl ester), with a stronger chemical attraction than the latter and to quench the fluorescence. The decrease in fluorescence of Fura-2 can reflect extracellular Mn^2+^ influx into cells. The determination of Ca^2+^ entry can be quantified the Mn^2+^-quenching technique (Ferreira et al., 2009). The Mn^2+^-quenching experiments were carried out by using the same equipment as that for intracellular Ca^2+^ measurements as described above. In the experiments, we used 4mM Mn^2+^ in the measurement buffer which corresponds to more than twice extracellular Ca^2+^ concentration. The fluorescence was excited at the isosbestic point at 360nm, and emission was monitored at 510nm. The rate of Mn^2+^ entry can be obtained from the slope of the fluorescence intensity curve with time. The rate of Mn^2+^-quenching is shown by the slope of the tangent against a quenching curve after the addition of stimulant. The basal rate of Mn^2+^-quenching can be determined by measuring “slopes 1” of the initial Fura-2 fluorescence decline, while the rate of fluorescence intensity decreases after the addition of TG (=“slope 2”). Therefore, ∆slope was regarded as an index to access the rate of fluorescence quenching by extracellular Mn^2+^ influx into cells.

### *In vitro* Stimulation and Proliferation Assays

Lymphocytes were prepared as described above, and cell proliferation was monitored using the cell tracker dye carboxyfluorescein diacetate succinimidyl ester (CFSE, Molecular Probes, Eugene, OR, United States) according to the standard procedure provided by Quah and Parish (2010). Briefly, cells were stained with CFSE dye at 5μM concentrations and loaded at 37°C for 10min, and the reaction was terminated by using PBS with 10% (v/v) heat-inactivated FCS. After washing, cells were resuspended in Dulbecco’s modified eagle medium (DMEM, PAA Laboratories, Pasching, Austria) with 10% (v/v) heat-inactivated FCS, 50mg/ml streptomycin, and 50U/ml penicillin. And the cells were seeded at a density of 2×10^6^ cells/per culture dish and then left to incubate at 37°C in a humidified 5% CO_2_ atmosphere with additional doses of 2.5μg/ml Con A or PHA (Sigma-Aldrich, St.Louis, MO, United States). After 72h of incubation, cells were harvested, stained with anti-CD3-PE antibody (Immunotools, Friesoythe, Germany). Data analysis was performed using a flow cytometer with FlowJo software (Version X; TreeStar, Ashland, OR, United States). AUC is defined as the area under the curve enclosed by the coordinate axis, of which the *x*-axis indicates cellular generations, and the *y*-axis shows the percentage of cells in each generation. △AUC as an index was used to evaluate cell proliferative ability.

### Quantitative Real-Time PCR

Murine CD3+ T cells were purified directly from splenic cells using an EasySep™ Mouse T Cell Isolation Kit (StemCell Technologies, Vancouver, CA, United States) based on magnetic bead separation technique according to manufactures’ instructions. Total RNA was extracted from T lymphocytes using the RNA isolation kit (RNeasy Mini Kit, Qiagen, Hilden, Germany), and a UV-Vis spectrophotometer (ND-1000, Nano-Drop Technologies, Rockland, United States) was used to determine the quantity and purity of the extracted RNA. To remove possible DNA contamination, on-column DNase digestion was applied by using the RNase-free DNase set (Qiagen, Hilden, Germany) in the context of RNA isolation. cDNA was subsequently synthesized by using a cDNA synthesis kit (high-capacity cDNA reverse transcription kit, Applied Biosystems) from RNA samples according to the manufacturer’s protocol. cDNA was obtained by on a PCR thermal cycler (T100, Bio-Rad Laboratories, Munich, Germany), and the product was used for qPCR. To determine mRNA expression in T cells, PCR was performed by using the iQ SYBR Green Supermix (Bio-Rad Laboratories, Munich, Germany), and an iCycler (Bio-Rad Laboratories, Munich, Germany) was applied to quantify the amplification products. The PCR primers used are listed in Table 1. The reaction conditions were as follows: 1cycle at 95°C for 3min, 42cycles at 95°C for 15s (denaturation), 61°C for 30s (annealing), and 72°C for 30s (elongation). The transcriptional level was normalized to the mRNA expression of housekeeping gene, β-actin. The results were calculated using the 2^−Δ(ΔCt)^ method and expressed as fold change in comparison with controls.

**Table 1:** List of primers for quantitative PCR.

Gene	Symbol	Forward primer (5'-3')	Reverse primer (5'-3')
Stromal interaction molecule 1	STIM1	TGAAGAGTCTACCGAAGCAGA	AGGTGCTATGTTTCACTGTTGG
ORAI calcium release-activated calcium modulator 1	ORAI1	AACGAGCACTCGATGCAGG	GGGTAGTCATGGTCTGTGTCC
ORAI calcium release-activated calcium modulator 2	ORAI2	GACCAAGTACCAGTACCCTCA	GCAAACAGATGCACGGCTAC
Inositol 1,4,5-triphosphate receptor 2	IP3R2	CCTCGCCTACCACATCACC	TCACCACTCTCACTATGTCGT
ATPase, Ca^2+^ transporting, cardiac muscle, slow twitch 2	SERCA2	GAGAACGCTCACACAAAGACC	ACTGCTCAATCACAAGTTCCAG
ATPase, Ca^2+^ transporting, plasma membrane	PMCA1	TGAAGGAGCTGCGATCCTCTT	CTGTTCCTGCTCAATTCGACT
Ryanodine receptor 2, cardiac	RYR2	ATTATGAAGGTGGTGCCGTATCA	TTCCACTCCACGCGACTCTTA
Purinergic receptor P2X, ligand-gated ion channel, 7	P2X7	GCACCGTCAAGTGGGTCTT	CAGGCTCTTTCCGCTGGTA
Transient receptor potential cation channel, subfamily C, member 1	TRPC1	ATCATCGGCCAAAACGATCAT	GCAGCTAAAATAACAGGTGCGA
Transient receptor potential cation channel, subfamily M, member 5	TRPM5	CCTCCGTGCTTTTTGAACTCC	CATAGCCAAAGGTCGTTCCTC
Transient receptor potential cation channel, subfamily V, member 4	TRPV4	AAACCTGCGTATGAAGTTCCAG	CCGTAGTCGAACAAGGAATCCA
Transient receptor potential cation channel, subfamily V, member 6	TRPV6	GACCAGACACCTGTAAAGGAAC	AGACACAGCACATGGTAAAGC
Calcium channel, voltage-dependent, R type, alpha 1E subunit	Cav2.3	AAGACCCCAATGTCTCGAAGA	TGGAAGATGAACCCTAGAGCC
Mitochondrial calcium uptake 1	MCU	CTTAACACCCTTTCTGCGTTGG	AGCATCAATCTTCGTTTGGTCT
ATPase, Ca^2+^ transporting, type 2C, member 1	ATP2C1	ATTGTGTGCGTGAAGGAAAACT	AAATAAGCGTAAGTCCGCAGG
Calmodulin 1	Calm1	CAGCGCACAACGCAGGT	TTCAGCAATCTGCTCTTCAGTCAG
Potassium channel, subfamily K, member 5	Kcnk5	TCTTCATCGTGTGGGGTGTCC	ATAGGGCGTGGTAGTTGGCAC
Heat shock protein 1A	HSPa1a	CATCCTGATGGGGGACAAGT	GTGGAGTTGCGCTTGATGAG
β-actin	Actb	GTGACGTTGACATCCGTAAAGA	GCCGGACTCATCGTACTCC

### Statistical Analysis

Data are expressed as means±SEM. Shapiro–Wilkʼs test revealed that data were normally distributed; thus, statistical analysis was performed using one-way ANOVA for comparison between groups. *p*<0.05 was set as statistical significance. SPSS 20.0 software was used throughout.

## Results

### Effect of Acute Exercise on Basal Intracellular Ca^2+^ Level and Agonist-Induced Ca^2+^ Transients

**Figure 1:**
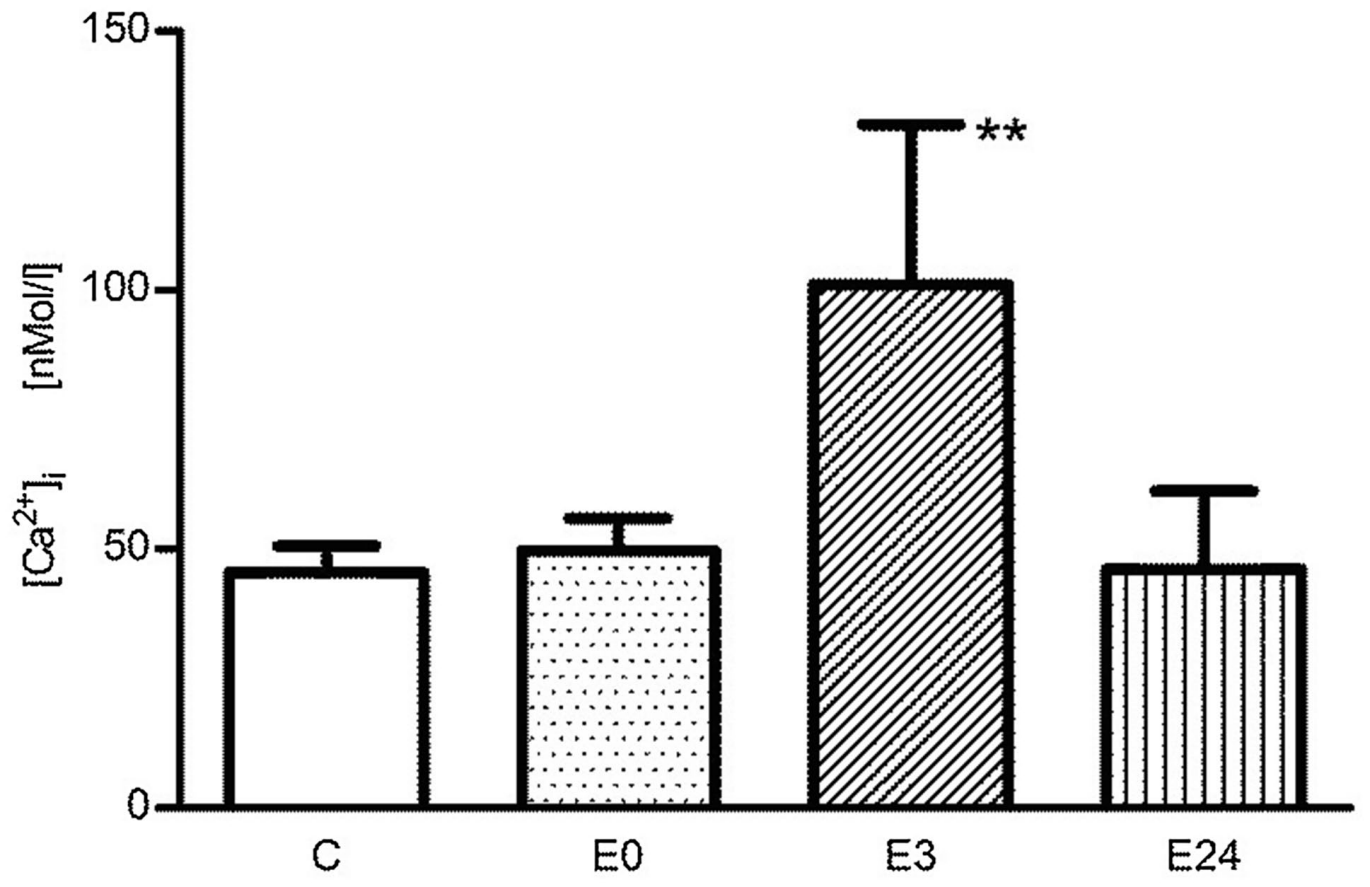
The change of basal [Ca^2+^]_i_ of murine splenic T lymphocytes in the control group (C), immediately after (E0), 3h after (E3), and 24h (E24) after acute exercise. Data are presented as means±SEM. ^**^p<0.01 vs. control.

In the control group, basal [Ca^2+^]_i_ of T cells was determined to be 45.5±5.1nM. Immediately after exercise, the resting [Ca^2+^]_i_ was 49.7±6.2nM, and no significant change was observed (*p*>0.01 vs. control, *n*=5). After 3h of post-exercise recovery, the resting [Ca^2+^]_i_ was highly significantly increased to 101.0±30.9nM (*p*<0.01 vs. control, *n*=5), and the resting intracellular calcium level 24h after exercise (E24) was 46.0±15.2nM and unchanged to pre-exercise conditions (*p*>0.01 vs. control, *n*=5; Figure 1).

**Figure 2:**
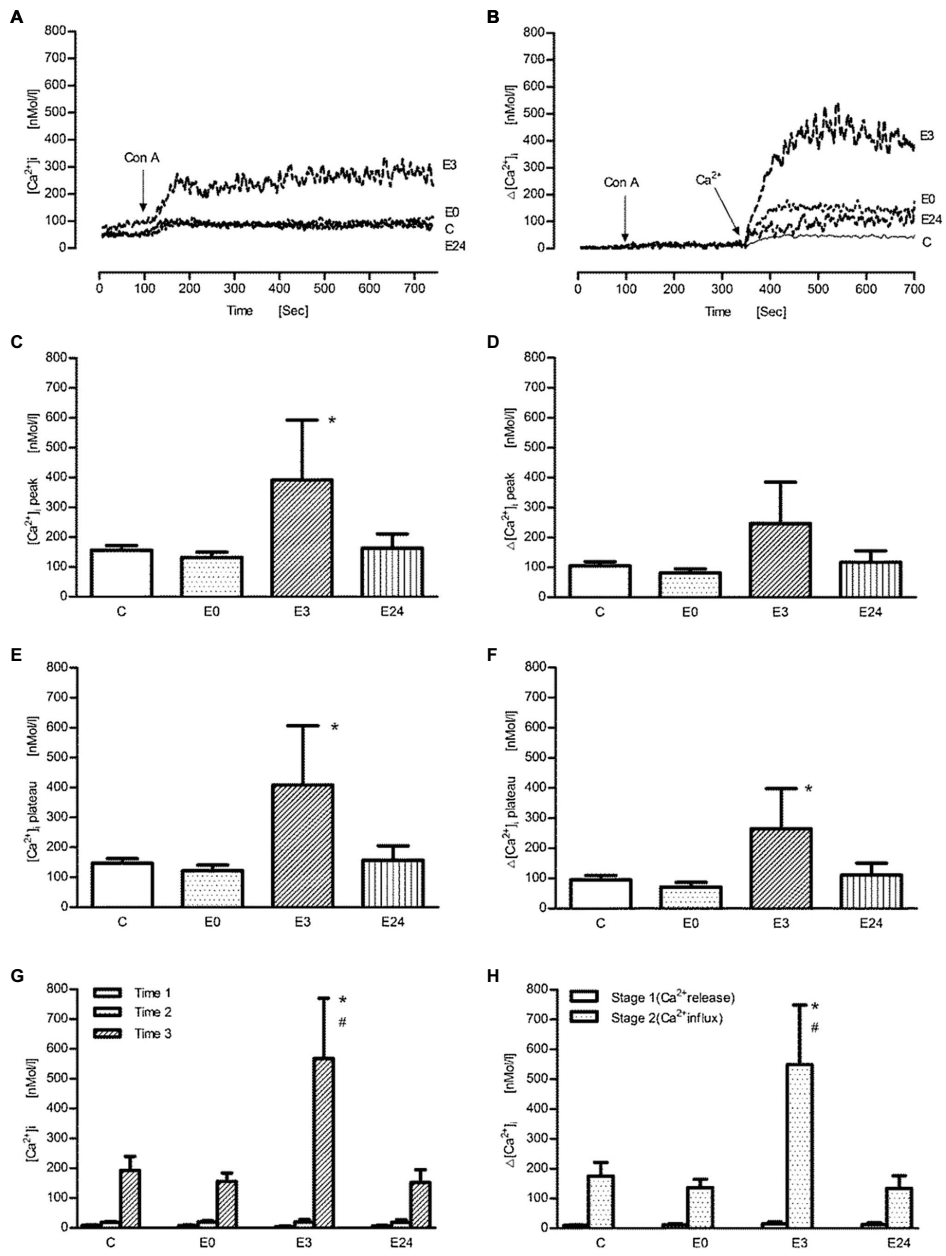
The effect of acute exercise on Con A-induced change of [Ca^2+^] of murine splenic lymphocytes at the different time points after exercise. **(A,B)** The representative tracings of Con A-induced change of intracellular Ca^2+^ signal in the calcium buffer and the Ca^2+^-free buffer, respectively; arrow shows when Con A (at 100s) or Ca^2+^ (at 350s) were applied; tracing C, the control group; tracings E0, E3, and E24 indicate the exercise groups with cell isolation at time points after, 3h, and 24h after exercise. **(C–H)** Bar chart diagrams summarizing the results of the entire groups. **(C–F)** The experiments were performed in the Ca^2+^ buffer; [Ca^2+^]_i_ and Δ[Ca^2+^]_i_ were referred to as absolute [Ca^2+^]_i_ and net [Ca^2+^]_i_ after the addition of Con A, respectively, and were presented separately for peak and plateau phase. **(G,H)** The measurements were performed in Ca^2+^-free buffer; Time 1: initial resting period, Time 2: addition of Con A, and Time 3: addition of CaCl_2_; the calculated Δ[Ca^2+^]_i_ levels of stage 1 and stage 2 indicate Ca^2+^ release from ER and transmembrane Ca^2+^ influx from extracellular space, respectively. Data are presented as means±SEM (*n*=5). ^*^ means *p*<0.05 vs. control; ^#^ means *p*<0.05 vs. E0.

Next, cells were stimulated with different agonists known to affect intracellular Ca^2+^ concentrations such as Con A and anti-CD3 antibody (OKT3). Representative tracings of intracellular Ca^2+^ signal after the administration of Con A in calcium containing or Ca^2+^-free buffer are shown in Figures 2A,B, respectively. The stimulant induced a biphasic intracellular Ca^2+^ transient with a peak and a plateau phase in calcium containing buffer. After addition of Con A, a significant absolute and net increase (=Δ[Ca^2+^]_I_) of intracellular Ca^2+^ concentration ([Ca^2+^]_i_) in the E3 group could be observed during both peak (Figures 2C,D) and plateau phase (Figures 2E,F; *p*<0.05 vs. control, *n*=5). In Figures 2G,H, the measurement was performed in the presence of Ca^2+^-free buffer. After an initial resting phase of 100s (=time 1), Con A was added (=time 2) followed by administration of CaCl_2_ (=time 3). By subtracting mean concentrations at time 1 from time 2 and at time 2 from time 3, the resulting differences should indicate Ca^2+^ release from intracellular stores (stage 1) and transmembrane Ca^2+^ influx (stage 2), respectively. After the administration of Con A, intracellular Ca^2+^ release wasnot affected in the E3 group (*p*>0.05 vs. control, *n*=5), while an improved transmembrane Ca^2+^ influx into cells could be observed (*p*<0.05 vs. control, *n*=5). Further stimulations were performed only up to 3h after exercise.

**Figure 3:**
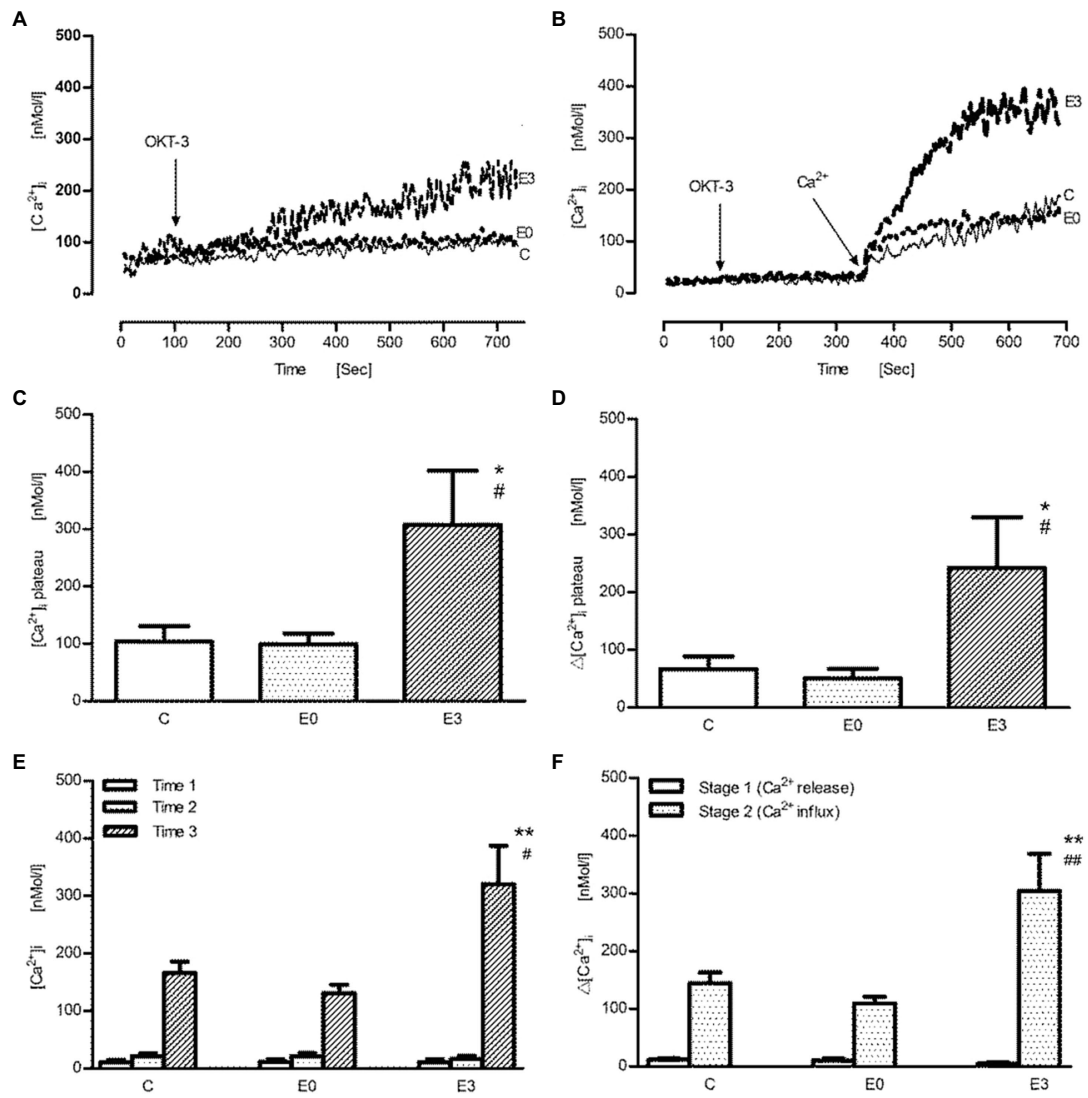
The effect of acute exercise on OKT3-induced change of [Ca^2+^]_i_ of murine splenic lymphocytes at the different time points after exercise. **(A,B)** The representative tracings of OKT3-induced change of intracellular Ca^2+^ signal in the calcium buffer and the Ca^2+^-free buffer, respectively; arrow shows when OKT3 (at 100s) and Ca^2+^ were applied (at 350s); tracing C, the control group; tracing E0, and E3 indicate the exercise groups with cell isolation at time points after and 3h after exercise. **(C–F)** Bar chart diagrams summarize the results of the entire group. **(C,D)** The measurement was performed in the Ca^2+^ buffer; [Ca^2+^]_i_ plateau and Δ[Ca^2+^]_i_ plateau were referred to as absolute [Ca^2+^]_i_ and net [Ca^2+^]_i_ during the plateau phase after stimulation with OKT3, respectively. **(E,F)** The measurement was performed in the Ca^2+^-free buffer; after the initiative scanning for time 1, OKT3 was added into the buffer for time 2. Next, calcium was administrated for time 3; the calculated Δ[Ca^2+^]_i_ levels of stage 1 and stage 2 indicate Ca^2+^ release from ER and transmembrane Ca^2+^ influx from extracellular space, respectively. Data are presented as means±SEM (*n*=5). ^*^p<0.05 or ^**^p<0.01 vs. control, ^#^*p*<0.05 or ^##^*p*<0.01 vs. E0 (*n*=5).

After stimulation with the anti-CD3 antibody (OKT-3), the representative tracings of intracellular Ca^2+^ signals in calcium containing or Ca^2+^-free buffer are shown in Figures 3A,B, respectively. In contrast to stimulation with Con A, a monophasic response after OKT-3 was found making an analysis of peak and plateau values quite impossible. The pattern of exercise effects on anti-CD3 induced Ca^2+^ transients, however, was similar to Con A. In Figures 3C,D, using 20μg/ml OKT-3, a significant increase of absolute and net [Ca^2+^]_i_ was found in the E3 group (*p*<0.05 vs. control, *n*=5). When cells were suspended first in Ca^2+^-free solution followed by addition of OKT3, the release of intracellular Ca^2+^ wasnot changed significantly in the E3 group (*p*>0.05 vs. control, *n*=5). Upon addition of Ca^2+^, an improved transmembrane Ca^2+^ influx was shown after exercise (*p*<0.01 vs. control, *n*=5; Figures 3E,F).

**Figure 4:**
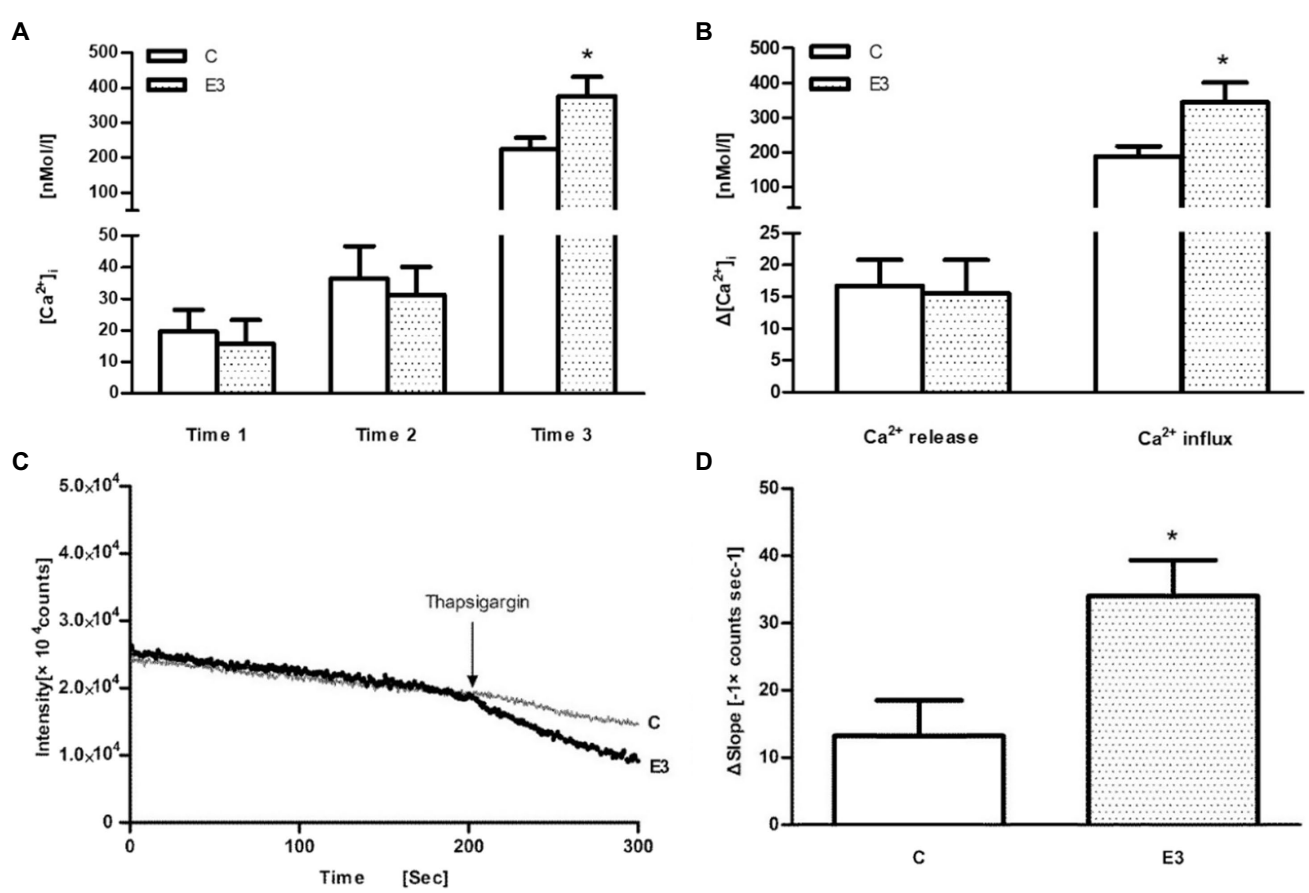
The effect of acute exercise on TG-induced change of [Ca^2+^]_i_ and Mn^2+^ influx in murine splenic lymphocytes. C: the control group; E3: the exercise group with cell isolation 3h after exercise. **(A)** Measurement of [Ca^2+^]_i_ initially under Ca^2+^-free buffer (=time 1), application of TG (=time 2), and addition of CaCl_2_ (=time 3); **(B)** Ca^2+^ release was quantified by the differences of levels between time 2 and time 1, whereas Ca^2+^ influx was quantified by the differences of levels between time 3 and time 2. **(C)** Representative tracings of the manganese influx measurements of the E3 group (heavy dotted line) and C group (light continuous line). **(D)** The bar chart diagrams show that transmembrane Mn^2+^ influx into cells after the addition of TG was statistically elevated in E3. Data are presented as means±SEM (*n*=5). ^*^p<0.05 vs. control (*n*=5).

Finally, thapsigargin (TG), an inhibitor of sarco-endoplasmic Ca^2+^-ATPase, was used. After application of TG in the Ca^2+^-free buffer, a similar release of Ca^2+^ from intracellular stores could be observed suggesting an identical load of intracellular calcium stores. However, after addition of Ca^2+^ into the extracellular medium, an enhanced Ca^2+^ influx signal was found in the E3 group suggesting an improved entry of extracellular calcium (*p*<0.05 vs. control, *n*=5; in Figures 4A,B). The calcium transients after TG stimulation were monophasic in nature (not biphasic as after Con A stimulation) and were analyzed therefore as single values.

### Effect of Acute Exercise in Transmembrane Mn^2+^ Influxes Into Cells

In order to confirm an exercise-associated enhanced Ca^2+^ entry, we used the Mn^2+^-quenching technique. As shown schematically in Figure 4C, the fluorescence intensity at a wavelength of 360nM continuously decreased after addition of Mn^2+^. After the application of TG, we observed a larger deflection of the Fura-2 fluorescence quenching in the E3 group (bold line) compared to control group (light line), which indicates an enhanced transmembrane Mn^2+^ influx into cells after exercise (*p*<0.05 vs. control, *n*=5), as shown in Figure 4D.

### Effect of Acute Exercise in Expression of Ca^2+^-Regulating Genes

**Figure 5:**
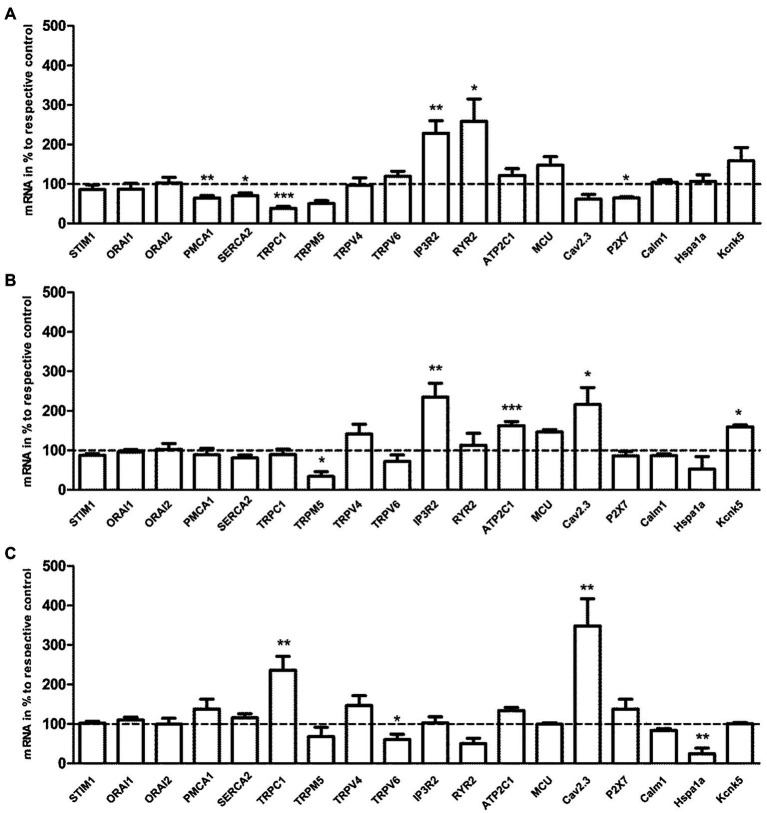
The effects of acute exercise in transcriptional level of Ca^2+^-regulating genes of murine splenic T lymphocytes. **(A)** mRNA expression of the E3 group vs. the control. **(B)** mRNA expression of the E24 group vs. the control. **(C)** mRNA expression of the E24 group vs. the E3 group. The transcription level of Ca^2+^-regulating genes was normalized to the housekeeping gene, β-actin mRNA. Note that columns and error bars represent (mRNA in % to respective control ±SEM); ^*^p<0.05, ^**^p<0.01, ^***^p<0.001 (*n*=5).

As shown in Figure 5, the transcription levels of cellular calcium ATPase (PMCA1, SERCA3) and ion channels (TRPC1, P2X7) were statistically reduced, while the transcription levels of internal Ca^2+^ release channels (IP3R2, RYR2) were significantly enhanced in the E3 group compared to control group (*p*<0.05/*p*<0.01 vs. control, *n*=5). The transcription level of ion channel, TRPM5, was significantly reduced, while transcription levels of intracellular Ca^2+^-regulating factors, IP3R2, ATP2C1, Cav2.3, and Kcnk5, were significantly increased in the E24 group compared to control group (*p*<0.05/*p*<0.01/*p*<0.001 vs. control, *n*=5). The transcription levels of TRPC1 and Cav2.3 were significantly increased, while the transcription levels of intracellular Ca^2+^-regulating genes, TRPV6 and Hspa1a, were statistically reduced in the E24 group compared to E3 group (*p*<0.05/*p*<0.01 vs. the E3 group, *n*=5).

### Effect of Acute Exercise in T Cell Proliferation

**Figure 6:**
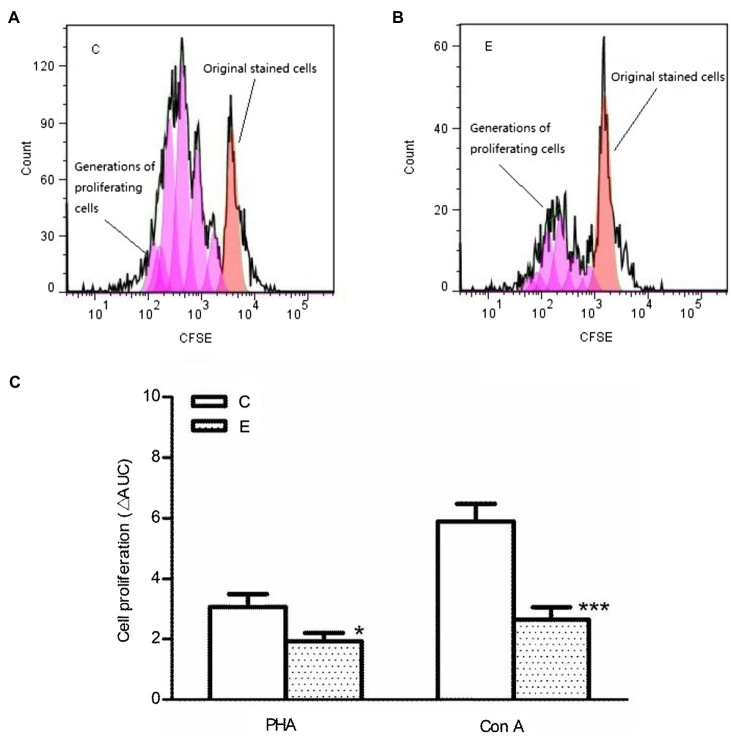
Effects of acute exercise on proliferative capacity of lymphocytes. **(A,B)** CFSE proliferation profile; **(C)** statistical analysis of cell proliferation. E, the exercise mice, which were killed 3h after exercise; C, the control group; Con A: concanavalin A; PHA: phytohemagglutinin. Data are presented as mean±SEM, ^*^p<0.05, ^***^p<0.001 vs. control (*n*=5).

A single acute bout of exhaustive exercise significantly reduced the mitogen-induced cell proliferation of T cells in the E3 group compared to control group (*p*<0.05/*p*<0.001 vs. control, *n*=5). The effect could be observed for both PHA and ConA, as shown in Figure 6.

## Discussion

The major findings of this study were that a single bout of intensive exercise caused reversible increases of both basal and agonist-induced intracellular Ca^2+^ concentrations during the early post-exercise recovery period. These changes could be related to an enhanced transmembrane Ca^2+^ influx but not to an altered load of intracellular calcium stores. However, enhanced Ca^2+^ transients were not transduced into an improved T cell function as indicated by an impaired cell proliferation suggesting the involvement of other intracellular signaling pathways.

The enhanced resting [Ca^2+^]_i_ after acute exercise might be the results of various underlying mechanisms. Exercise stress might damage cell membranes directly by mechanical stress as shown for foot-strike hemolysis during long distance running (Fazal et al., 2017). Changes in cell membrane integrity might result also from enhanced lipid peroxidation due to enhanced exercise-associated oxidative stress. Likewise, previous data from our group have proved that intracellular Ca^2+^ of lymphocytes was affected transiently by acute exercise in humans (Mooren et al., 2001). However, membrane damage seems to be unlikely in the actual case as it should occur very early during and after exercise. The delayed alteration of resting [Ca^2+^]_i_ (=3h post-exercise) in the current study points to another, more likely regulatory processes. The determination of Ca^2+^-handling proteins mRNA showed a significant reduced expression of both the plasma membrane Ca^2+^-ATPases (PMCA) and sarco/endoplasmic reticulum Ca^2+^-ATPases (SERCA). Ca^2+^-ATPase maintains the global basal free Ca^2+^ levels actively on the expense of ATP in order to transport free calcium ion against its electrochemical gradient either out of cells or into intracellular Ca^2+^ stores. The decreased expression of Ca^2+^-ATPases could mean a decreased enzyme activity and an impaired ability to regulate intracellular Ca^2+^ levels. The reduced Ca^2+^-ATPase expression and function should therefore result in a cytoplasmic Ca^2+^ buildup as a result of a new balance between Ca^2+^ increasing and decreasing processes. Previous studies showed also that the impairment in Ca^2+^ homeostasis just due to one of the Ca^2+^ extruding pathway is enough to interfere with calcium homeostasis and cell function. Therefore, defective Ca^2+^ extrusion due to decreased PMCA expression was responsible for an enhanced cell apoptosis (Pellegrini et al., 2007).

Azenabor and Hoffman-Goetz (2000) found that exhaustive exercise caused the increase of intracellular Ca^2+^ levels in murine thymocytes, and there was a continuous influx of Ca^2+^ after exercise when cells were monitored in Ca^2+^ rich medium. Intracellular Ca^2+^ level actually is the result of a mutual balance among Ca^2+^-increasing, Ca^2+^-decreasing, and Ca^2+^-buffering mechanisms (Mooren and Kinne, 1998). Exercise is obviously able to transiently disturb the balance among the Ca^2+^-regulating mechanisms in resting cells, which was fully reversible 24h after exercise. Beside the calcium handling machinery, however, other signaling pathways might be responsible for the dysregulation of basal [Ca^2+^]i after exercise. There is evidence that an activation of the transcription factor nuclear factor of activated T cells (NFAT), a key regulator of T cell activation, is followed by increased basal Ca^2+^ levels (Martinez et al., 2015).

Next, acute exercise was followed also by enhanced agonist-induced intracellular Ca^2+^ dynamics independent of the stimulant used (Con A, OKT3, and TG). In order to discriminate between the contribution of Ca^2+^ release from intracellular pools such as endoplasmic reticulum and Ca^2+^ influx *via* transmembrane Ca^2+^ channels such as store-operated calcium entry (SOCE), a unique plasma membrane Ca^2+^ entry mechanism, experiments were performed under Ca^2+^-free conditions. CRAC channels, which are formed by ORAI1-3, are the major SOCE channels in T cells; other channels, such as transient receptor potential (TRP) and voltage-dependent calcium (Cav) channels, also can participate in SOCE (Hoth, 2016). However, at least the voltage-gated Ca^2+^ (Cav) channels are less characterized in T cells. The results suggest that after exercise the transmembrane Ca^2+^ influx into T cells is the major factor for the altered Ca^2+^ signals. Likewise, thapsigargin (TG) by inhibiting the SERCA pumps releases similar amounts of Ca^2+^ from intracellular Ca^2+^ pools suggesting that their load wasnot affected by exercise. Furthermore, the Fura-2-quenching experiments using Mn^2+^ as a surrogate permeable bivalent cation for Ca^2+^ after application of TG support that intensive exercise could improve the transmembrane Ca^2+^ entry during the early (3h) post-exercise recovery period. The capacity of extracellular Ca^2+^ influx is thereby directly proportional to the ER store depletion (Zweifach and Lewis, 1996). The linearity of the capacity to deplete ER Ca^2+^ stores to determine the subsequent degree and timing of influx could offer an explanation for that the experiments performed in the calcium medium evoked a lower Ca^2+^ entry than those in the Ca^2+^-free medium. Another explanation might be the nonlinear response of the fluorescent dye especially during/after Ca^2+^-depleted conditions.

A challenging question is how alterations of Ca^2+^-handling factors fit with the changed Ca^2+^ transients. The downregulated expression of Ca^2+^-extruding pumps should delay Ca^2+^ extrusion resulting in enhanced Ca^2+^ transients as observed. The individual contribution of the Ca^2+^-ATPase is exquisitely coordinated in T cells in time and space in order to effectively modulate intracellular Ca^2+^ dynamics upon activation. PMCA has previously been shown to functionally associate with SOCE channels (Bautista et al., 2002). Thus, Ca^2+^ entry pathways such as TRPC1 and P2X7 have been downregulated as well. The role of purinergic ionotropic P2X7R in T cells has been studied in mice; the activation requires millimolar concentrations of its ligand ATP, which can induce the influx of Ca^2+^/Na^+^ and the efflux of K^+^ (Foster et al., 2013). The expression of the TRPV4, a Ca^2+^-permeable nonselective ion channel that is sensitive to variations of body temperature has been shown to be affected by chronic exercise in a tissue dependent manner (Chen et al., 2015). However, acute exercise as used in the actual study did not affect its expression in T cells. It might be speculated that the decreased expression of some transmembrane Ca^2+^ channels serves as a protective mechanism to prevent a further intracellular Ca^2+^ overload. In contrast, the enhanced expression of both IP3R2 and RYR2 receptors had no effects on the calcium load of intracellular stores, but it may have an amplifying effect during agonist induced Ca^2+^ transients. In T cells, Ca^2+^ release from the ER is mediated by binding of IP3 to IP_3_R and is further modulated by RyR (Dadsetan et al., 2008). For a proper function, SOCE requires the assembly of ER-located STIM proteins with the plasma membrane channels which occurs within distinct regions in the cell that have been termed as endoplasmic reticulum (ER)–plasma membrane (PM) junctions. The PM and ER are in close proximity to each other within this region, which allows STIM1 in the ER to interact with and activate either ORAI1 or TRPC1 in the plasma membrane (Subedi et al., 2017). Higher expression of intracellular Ca^2+^ release channels might therefore modulate Ca^2+^ permeability and enhance transmembrane Ca^2+^ influx despite lower expression of Ca^2+^ entry pathways. Compared to our recent publication about the effects of regular long-term exercise training on T cell and Ca^2+^ signaling, similarities and dichotomies were found. Exercise training had no effect on the expression of Ca^2+^ extruding pumps from both cell membrane and intracellular stores. Ca^2+^ channel proteins from cell membrane and intracellular stores, such as ORAI1, IP3R2, TRPM5, TRPC1, and TRPV4 Ca2.3 were broadly downregulated (Liu et al., 2017). In contrast, acute exercise induced upregulation of some Ca^2+^ channels such as the intracellular channels IP3R2 and RYR2 and some transmembrane Ca^2+^ channels like Cav2.3 and TRPC1 suggesting a differential regulation of Ca^2+^ signaling in T cells by acute and chronic exercise. The enhanced expression of Cav2.3 occurred delayed 24h after exercise without any influence on cellular Ca^2+^ signals. Recently, the Cav2.3 gene was shown to be upregulated in pregnancy and type 1 diabetes indicating a shift in immunological status under these conditions (Bhandage et al., 2018).

What might be the exercise-associated condition which modulates transiently intracellular calcium? Previous work has demonstrated that levels of oxidative stress are increased after intensive bouts of exercise (Kruger et al., 2009). Moreover, it has been well documented that free radicals are able to modulate the activity of many Ca^2+^-handling proteins (Gorlach et al., 2015), which may result in changed capacitative Ca^2+^ entry and finally in intracellular Ca^2+^ overload. The function of PMCA can be altered by an enhanced lipid peroxidation (Kako et al., 1988; Ohta et al., 1989), and SERCA is highly susceptible to oxidative damage, which can lead to a decrease of SERCA activity and then result in elevation of intracellular Ca^2+^ level (Antipenko and Kirchberger, 1997; Squier and Bigelow, 2000). On the other hand, the elevation of cytosolic Ca^2+^ level can activate the mitochondrial Ca^2+^ uniporter (MCU) that induces mitochondrial Ca^2+^ uptake, an event that drives mitochondrial respiration thereby causing the generation of further free radicals, which may amplify the cellular oxidative stress. A Ca^2+^ overload in mitochondrial matrix causes the opening of the permeability transition pore (PTP) followed by a collapse of mitochondrial potential, mitochondrial swelling and release of cell death inducing agents (Gherardi et al., 2020). Such sequence finally leads to apoptotic cell death, which recently was described to be induced after exhaustive exercise (Kruger et al., 2009).

Finally, acute exhaustive exercise can modulate the ability of T cell proliferation. Many studies have shown that an acute bout of strenuous exercise is followed by reduced immune cells functions in humans and mice (Randall Simpson et al., 1989; Mazzeo et al., 1998; Potteiger et al., 2001). The results of this study confirmed these results in the murine model by demonstrating a reduced proliferative response of splenic lymphocytes to mitogens. However, after acute exercise, the increased intracellular Ca^2+^ signals were not transferred into an enhanced cellular function contrary to our recent findings after chronic exercise training. Chronic moderate exercise elevated basal Ca^2+^ level and agonist-induced Ca^2+^ influx, which was followed by an improved lymphocyte proliferation (Liu et al., 2017). These contrary findings suggest that after acute exercise the usual stimulus–response relationship is broken at least for cell proliferation as one of several T cell functions. One reason might be that exercise induces alterations of spatial and temporal Ca^2+^ patterns such as calcium oscillations, which could not be recognized using the actual technical approach. The increasing [Ca^2+^]_i_ *per se* is not enough to trigger cell proliferation (Silberman et al., 2005). The Ca^2+^ signaling promotes cellular proliferation depending on the amplitude of increase, frequency of oscillations, the duration and nature of change, and the location of cytosolic Ca^2+^ responses (Monteith et al., 2007; Brini and Carafoli, 2009). On the other hand, cellular proliferation ability is controlled also by other signaling pathways than [Ca^2+^]_i_, such as transcription factors like cAMP response element-binding protein (CREB), AP-1, and NFAT (Dolmetsch et al., 1998; Martinez et al., 2015; Shin et al., 2019). T cells show hyporesponsive states during conditions of persistent stimulation, referred to as cellular exhaustion. There is evidence that such a hypo-response does not affect all cellular functions to the same degree. Moreover, impaired cellular proliferation occurs despite preserved intracellular Ca^2+^ signaling (Agnellini et al., 2007). NFAT could be identified as a major factor responsible for promoting exhaustion of activated T cells (Martinez et al., 2015). Further studies should therefore focus on the exercise-dependent regulation of cellular transcription factors. Another limitation of the current study includes the investigation of cell suspensions and not single cells. The latter approach would enable the detection of individual calcium signals and patterns and the use of the patch clamp technique for electrophysiological measurements. With the development of molecular biology cloning technology, we have gradually learned that there are great similarities of molecular signatures between humans and animals, but there are also certain differences. Although it is tempting to extrapolate results from one species to another, doing such without assessing the differences of the various ion channels, such as calcium and potassium channels, in different species brings risks, as translation is not always that simple (Tanner and Beeton, 2018).

Thus, taken together, our findings demonstrated that acute exercise affects intracellular calcium homeostasis during resting and stimulated conditions in murine T cells. The changed calcium levels result from an enhanced transmembrane Ca^2+^ influx into cells and an altered expression of cellular Ca^2+^ ATPases such as PMCA and SERCA as well as intracellular Ca^2+^ release channels such as IP3R and RyR. However, the alterations of Ca^2+^ signaling could not be related to the inhibition of cell proliferation in T cells making further studies on the role of additional signaling pathways such as transcription factor activation necessary.

## Data Availability Statement

The original contributions presented in the study are included in the article/supplementary material, further inquiries can be directed to the corresponding author.

## Ethics Statement

The animal study was reviewed and approved by the Animal Care and Use Committee of Giessen University.

## Author Contributions

All authors have made substantial contributions to the conception or design of the work or the acquisition, analysis, or interpretation of data for the work, drafted the work or revised it critically for important intellectual content, approved the final version to be published, and agreed to be accountable for all aspects of the work in ensuring that questions related to the accuracy or integrity of any part of the work are appropriately investigated and resolved. All authors contributed to the article and approved the submitted version.

## Conflict of Interest

The authors declare that the research was conducted in the absence of any commercial or financial relationships that could be construed as a potential conflict of interest.

## Publisher’s Note

All claims expressed in this article are solely those of the authors and do not necessarily represent those of their affiliated organizations, or those of the publisher, the editors and the reviewers. Any product that may be evaluated in this article, or claim that may be made by its manufacturer, is not guaranteed or endorsed by the publisher.
